# Copy Number Variations of the *NSMF* Gene and Their Associations with Growth Traits in Three Chinese Sheep Breeds

**DOI:** 10.3390/genes16020218

**Published:** 2025-02-13

**Authors:** Xiukai Cao, Yongqi Liu, Jie Cheng, Chen Ling, Jinlin Huang, Wei Sun

**Affiliations:** 1 Joint International Research Laboratory of Agriculture and Agri-Product Safety of Ministry of Education of China, Yangzhou University, Yangzhou 225009, China; cxkai0909@163.com; 2 College of Animal Science and Technology, Yangzhou University, Yangzhou 225009, China; 3 Jiangsu Key Laboratory of Sericultural and Animal Biotechnology, Jiangsu University of Science and Technology, Zhenjiang 212100, China; 4 Jiangsu Key Laboratory of Zoonosis, Yangzhou University, Yangzhou 225009, China

**Keywords:** sheep, *NSMF*, CNV, growth traits, association

## Abstract

Background/Objectives: Copy number variations (CNVs) are a significant source of genetic variation and have been shown to influence growth traits in livestock. This study aimed to validate previous CNV candidates within the *NSMF* gene (XM_015093798.1) and identify novel CNV markers for molecular breeding in sheep. Methods: Using quantitative PCR (qPCR), we genotyped *NSMF* CNVs (chr3: 586,001–601,000) and assessed their associations with growth traits in three Chinese sheep breeds: Chaka sheep (CKS, *n* = 312), Hu sheep (HS, *n* = 67), and Small-tailed Han sheep (STHS, *n* = 70). Results: Our results revealed significant differences in *NSMF* CNV genotype frequencies across the three breeds, with the highest proportion of deletions observed in STHS (98.44%) and CKS (90.57%), while HS exhibited a higher frequency of duplications (14.06%). No significant associations were observed between *NSMF* CNV genotype and CKS growth traits (*p*-value > 0.05). However, the CNV could markedly affected cannon circumference in HS (*p*-value = 0.021), with individuals carrying the normal genotype showing a larger cannon circumference. Additionally, a marginally significant association was found between the CNV and body diagonal length in HS (*p*-value = 0.050). Conclusions: Future investigations employing larger cohorts of Hu sheep are warranted to definitively establish the utility of *NSMF* CNVs as genetic markers for growth traits in Hu sheep breeding programs.

## 1. Introduction

Sheep (*Ovis aries*) play a crucial role in global agriculture, providing valuable products such as meat, milk, and wool. Improving important economic traits through selective breeding is a primary goal for sheep breeders. The identification and utilization of molecular markers associated with growth traits can significantly accelerate the breeding process and enhance selection accuracy [1].

Copy number variations (CNVs), a type of structural variation in the genome, refer to alterations in DNA segments ranging from kilobases to megabases, resulting in abnormal deletions, insertions, duplications, and complex rearrangements compared to a reference genome [2]. The advancement and maturity of microarray and sequencing technologies have fueled a substantial increase in CNV research. To date, most livestock and poultry species, including pig, cattle, buffalo, yak, sheep, goat, horse, donkey, and chicken, have had their CNVs discovered [3,4,5]. CNVs are widespread in animal genomes and are recognized as a significant source of genetic variations. Using next-generation sequencing data, researchers identified 24,558 putative CNVs spanning 197 Mb, corresponding to approximately 6.9% of the sheep genome, in a worldwide population of 2254 sheep representing 68 breeds [6]. In recent years, increasing evidence has demonstrated the important roles of CNVs in regulating gene expression, affecting protein function, and contributing to complex traits in humans and livestock [7]. It was reported that dosage sensitivity is a major determinant of human copy number variant pathogenicity [8,9]. A genome-wide association study based on CNVs identified various CNVRs significantly associated with feed efficiency and growth traits in Nellore cattle [10]. While using quantitative PCR (qPCR), the CNV of candidate gene *MICAL-L2* (molecule interacting with casL-like 2) was shown to shape gene expression and contribute to different phenotypes in four Chinese cattle breeds [11]. Interestingly, in a case–control analysis (thin-tailed sheep vs. fat-tailed sheep), a copy-gained CNVR harboring *HGFAC* (hepatocyte growth factor activator) and *LRPAP1* (low-density lipoprotein-related protein-associated protein 1) genes was found to be associated with fat deposition [12]. These findings reinforce the belief that CNVs can serve as important targets for molecular breeding in livestock and complex human diseases.

In our previous study of 412 sheep from 62 major global domestic sheep breeds and wild relative species, we identified 24,534 CNVRs (average length of 3584 bp; total length of 87.92 Mb); however, most of these variations have not been validated and subjected to association analysis [13]. Among these CNVRs, eight overlap *NSMF* (N-methyl-D-aspartate receptor synaptonuclear signaling and neuronal migration factor), a promising candidate gene for sheep growth traits. The *NSMF* gene encodes the Jacob protein, a crucial synaptonuclear messenger involved in diverse cellular processes [14]. Jacob transduces signals from synaptic and extrasynaptic NMDA (N-methyl-D-aspartate) receptors to the nucleus, assembling distinct signalosomes that dock to the transcription factor CREB (cAMP-response element-binding protein), thereby modulating gene expression [15]. Beyond its role in neuronal signaling, *NSMF* has been identified as a key factor in the DNA damage response, specifically promoting ATR (ataxia telangiectasia and rad-3-related kinase) activation and RPA (replication protein A) phosphorylation upon replication stress, thus ensuring genome maintenance [16,17]. Furthermore, recent research has also implicated *NSMF* in myoblast proliferation, suggesting a novel role in muscle development and potential therapeutic applications [18]. These findings highlight the multifaceted roles of *NSMF* in neuronal signaling, DNA damage response, development, and potentially muscle function.

All the above facts prompt us to investigate the potential association between *NSMF* CNVs and growth traits in sheep. Therefore, in this study, we used quantitative PCR (qPCR) to validate the presence of *NSMF* CNVs previously identified, to determine the copy number of *NSMF* gene in each individual, and to analyze the associations between *NSMF* CNV types and growth traits of three important Chinese sheep breeds, Chaka sheep (CKS), Hu sheep (HS), and Small-tailed Han sheep (STHS). Identifying genetic markers associated with desirable growth traits is crucial for improving breeding efficiency and productivity in sheep. Therefore, the findings of this study aim to provide novel insights into the genetic architecture of growth traits and potentially identify valuable molecular markers for implementation in sheep breeding programs, ultimately contributing to enhanced genetic selection and livestock improvement.

## 2. Materials and Methods

### 2.1. Animals and Phenotype Data Collection

**Figure 1:**
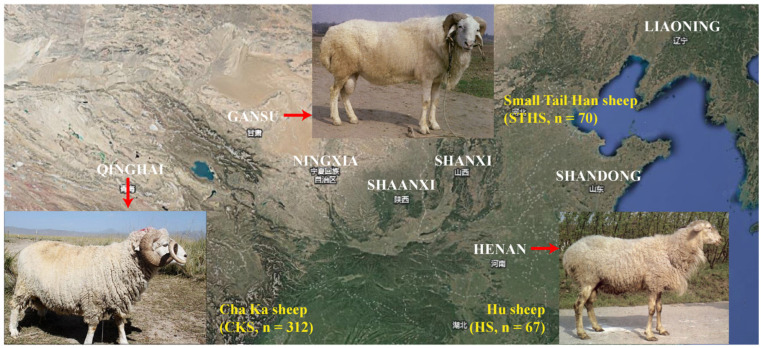
Distribution and sample sizes of three Chinese sheep breeds.

This study included three important Chinese sheep breeds, Chaka sheep (CKS, *n* = 312), Hu sheep (HS, *n* = 67), and Small-tailed Han sheep (STHS, *n* = 70) (Figure 1). CKS is a wool–mutton-type semi-fine-wool breed; HS and STHS are both dual-purpose breeds utilized for both meat and pelt production. The CKS utilized in this study were all adults (1~6 years old) and were obtained from Chaka Town, Haixi Prefecture, Qinghai Province. The group consisted of 112 ewes, 90 rams, and 110 castrated rams. HS (67 ewes, 8 months old) were obtained from Henan Shanshan Agricultural and Pastoral Technology Co., Ltd. (Mengjin County, Henan Province, China). STHS (42 ewes, 28 rams, 1~2 years old) were obtained from Ruilin Technology Breeding Co., Ltd. (Yongjing County, Gansu Province, China).

The sheep were raised under standard, semi-intensive management conditions. They were housed in open-sided sheds with access to pasture. They were provided with a balanced diet consisting of pasture grazing supplemented with hay and a commercial concentrate feed. Water was available ad libitum. The samples used in this study were collected from non-pregnant, non-lactating ewes. The growth data collection for this study took place during the autumn season. Growth traits were measured according to previous reports [19,20]. Withers height, body length, chest girth, and body weight were recorded for CKS; withers height, pin bone width, body diagonal length, chest girth, cannon circumference, and body weight were recorded for HS; and withers height, chest depth, chest girth, cannon circumference, and rump height were recorded for STHS. All sheep were selected to minimize relatedness within the past three generations.

### 2.2. DNA Extraction

This study did not involve any invasive procedures beyond routine blood sampling, and no animals were euthanized for the purposes of this research. Genomic DNA was extracted from 2.0 mL of jugular vein blood of each animal according to phenol–chloroform method as described previously [21]. Extracted DNA was assessed for concentration and quality using a NanoDrop 2000 spectrophotometer (Thermo Fisher Scientific, Waltham, MA, USA) and subsequently diluted to 25 ng/μL before being stored at −20 °C until further use.

### 2.3. Quantitative PCR Analysis for NSMF CNV Detection

Quantitative PCR (qPCR) was performed to validate the presence and determine the copy number of the *NSMF* CNVs previously identified (Oar_v4.0/oviAri4, chr3: 586,001–601,000) [13]. Primers specific to the *NSMF* CNV were designed using Primer Premier 5.0 software. The *ANKRD1* (ankyrin repeat domain 1) gene was employed as a reference gene, predicated on the general consensus that it is not subject to copy number variation [22]. The primer sequences were listed in Table 1.

Melting curve analysis was performed to confirm primer specificity, and no-template control reactions were included to detect potential contamination. qPCR reactions were performed in triplicate. Each 12.5 μL reaction mixture contained 25 ng of DNA, 6.25 μL of 2× SYBR Green qPCR Mix (Aidlab, Beijing, China), 1 μL 10.0 pmol of primer pair, and 4.25 μL of nuclease-free water. Thermal cycling conditions consisted of an initial denaturation at 94 °C for 4 min, followed by 39 cycles of denaturation at 94 °C for 10 s, annealing and extension at 60 °C for 60 s, and a final extension/melting curve analysis at 95 °C for 10 s.

### 2.4. Determination of NSMF Gene Copy Number

The *NSMF* gene copy number was determined using the 2 × 2^−ΔCt^ method. The ΔCt value was calculated by subtracting the average Ct value of the reference gene from the average Ct value of the *NSMF* gene. The values of 2 × 2^−ΔCt^ were rounded to the nearest whole number. Samples were classified into three categories: deletion (copy number < 2), normal (copy number = 2), and duplication (copy number > 2).

### 2.5. Statistical Analysis

Differences in CNV frequencies among the three breeds were assessed using the chi-square test of R software (version 4.3.1). The associations between *NSMF* CNV genotype and sheep growth traits of each breed were analyzed using a general linear model in SPSS software (version 23), with CNV genotype and sex as fixed effects [23]. Age was used as a random effect only for CKS analysis. *p*-values less than 0.05 were considered statistically significant.

## 3. Results

### 3.1. NSMF Gene CNVs and Validation of Detection Primers

**Figure 2:**
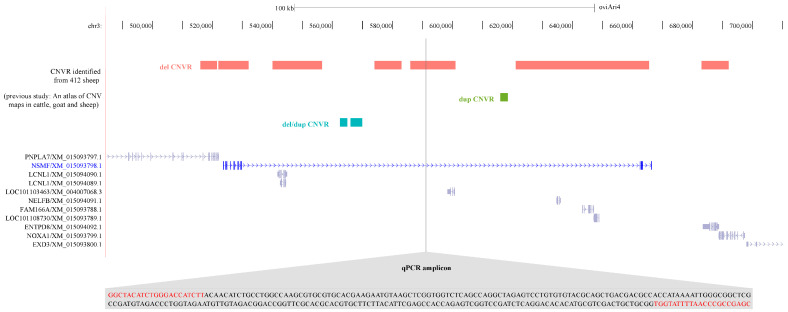
Previously characterized *NSMF* CNVs and primer target sites in this study.

Our previous research identified eight potential copy number variable regions (CNVRs) within the *NSMF* gene (chr3:523,546–666,320). These included five deletion CNVRs, one duplication CNVR (412 sheep; del: 0; dup: 378), and two mixed CNVRs (412 sheep; del: 63; dup: 26; del: 152; dup: 10) exhibiting both deletion and duplication events. For this study, we focused on a deletion CNVR (412 sheep; del: 315; dup: 4) located within the central region of the *NSMF* gene (Oar_v4.0/oviAri4, chr3: 586,001–601,000) (Figure 2).

**Figure 3:**
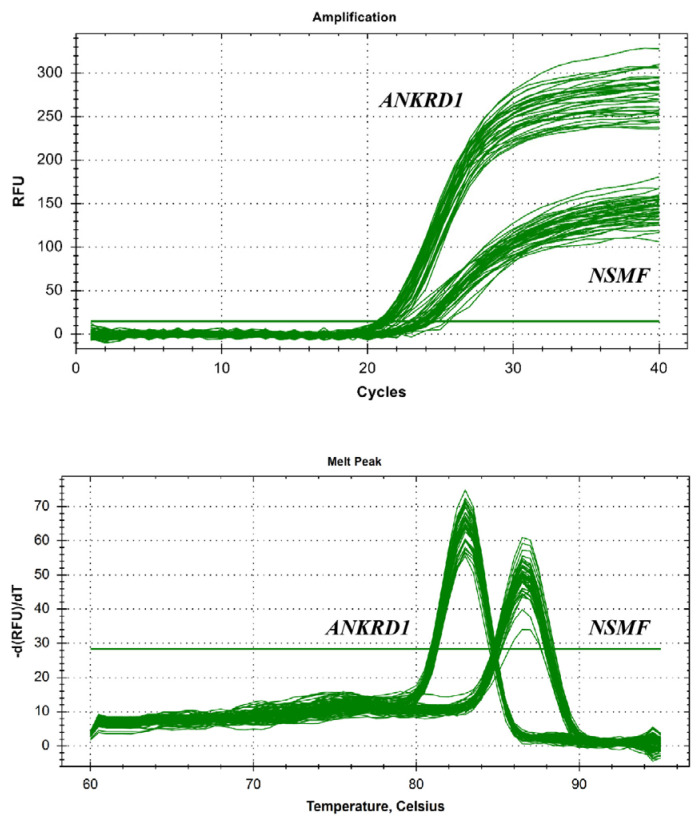
Amplification and melting curves generated by primers targeting the selected *NSMF* CNVR (top and bottom panels).

To assess the effectiveness of primers designed for the target gene *NSMF* CNV and the reference gene *ANKRD1*, qPCR was performed using DNA from three sheep breeds representing diverse genetic backgrounds and exhibiting distinct growth characteristics as templates. As shown in Figure 3, the melting temperatures of the amplification products from both primer pairs were between 80 °C and 90 °C, and the melt peaks were all single peaks without any extraneous peaks. The Ct values were all between 20 and 30 cycles. These results indicate that the primers for *NSMF* CNVs and the reference gene *ANKRD1* are effective and can be used for subsequent experiments.

### 3.2. NSMF CNV Profile and Genotype Frequencies in Three Sheep Breeds

**Figure 4:**
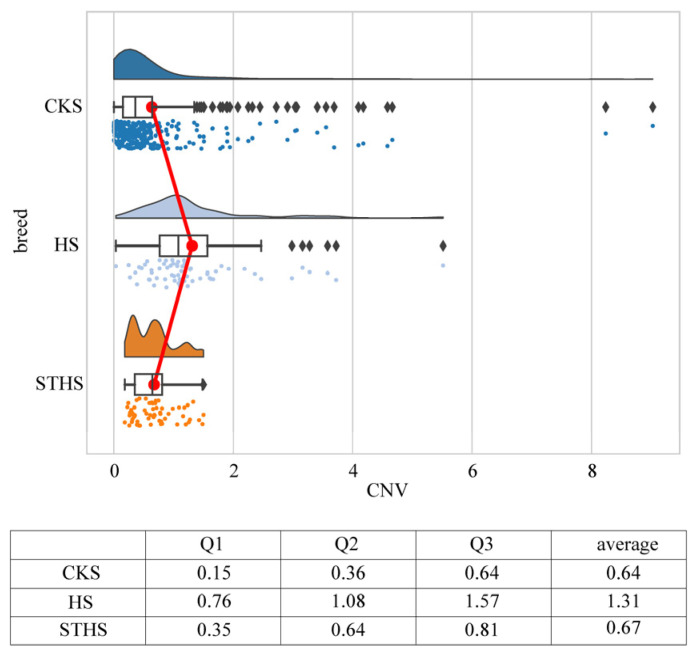
*NSMF* gene copy number variation in three Chinese sheep breeds. Red line, the trend of average copy number variation; black diamonds, individuals with abnormal copy numbers; colored dots, the copy number of the *NSMF* gene for each individual.

Using the 2 × 2^−ΔCt^ method, we calculated the copy number of *NSMF*. As shown in Figure 4, the *NSMF* copy number in the CKS breed ranged from 0 to 9, with an average copy number of 0.64. The first quartile (Q1) was 0.15, the second quartile (Q2), or median, was 0.36, and the third quartile (Q3) was 0.64. In the HS breed, the copy number ranged from 0.03 to 5.51, with an average copy number of 1.31. The Q1 was 0.76, the Q2 was 1.08, and the Q3 was 1.57. In the STHS breed, the copy number ranged from 0.18 to 1.50, with an average copy number of 0.67. The Q1 was 0.35, the Q2 was 0.64, and the Q3 was 0.81.

**Figure 5:**
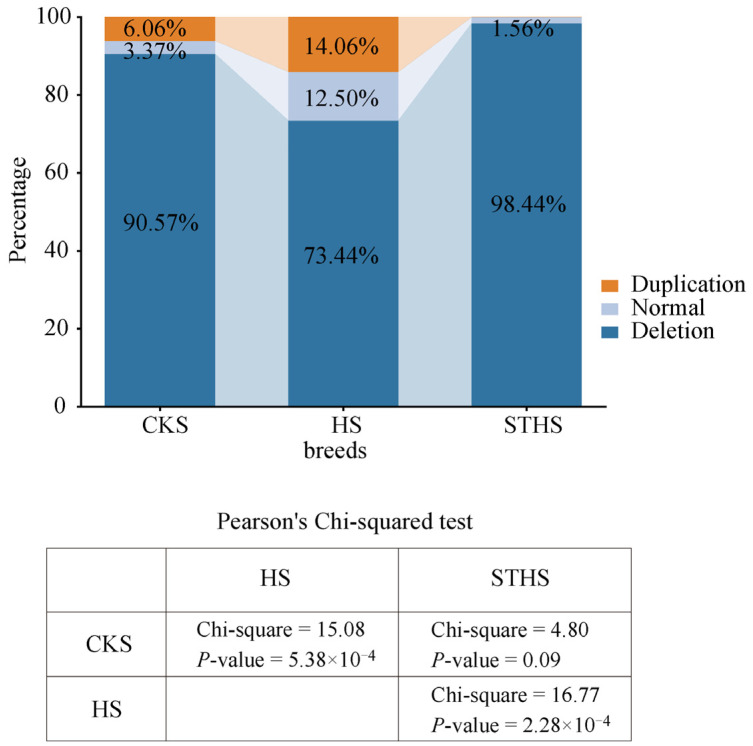
Comparative analysis of *NSMF* genotype frequencies in three Chinese sheep breeds.

Furthermore, based on the rounded results of 2×2^−ΔCt^, we classified the *NSMF* CNV into three genotypes: deletion (copy number < 2), normal (copy number = 2), and duplication (copy number > 2). The results showed that the deletion of the *NSMF* CNV had the highest proportion in all three sheep breeds: 90.57% in CKS, 73.44% in HS, and 98.44% in STHS (Figure 5). These results are consistent with our previous identification of *NSMF* CNV as a deletion. Among the three sheep breeds, the HS breed had the highest proportion of *NSMF* CNV duplication (14.06%), while the STHS breed had the lowest (0%). Pearson’s chi-square test revealed significant differences in the genotype frequencies of *NSMF* CNV among the three sheep breeds, with the exception of no significant difference between CKS and STHS (chi-square = 4.80, *p*-value = 0.09).

### 3.3. Association Analysis of NSMF CNV with Growth Traits in Sheep

Based on the *NSMF* CNV genotyping results, we performed association analyses to identify potential molecular markers for sheep molecular breeding. Due to the low *NSMF* CNV polymorphism in STHS, with only one normal individual and no duplication individuals, association analysis could not be conducted for this breed. Therefore, we performed association analyses between growth traits and *NSMF* CNV in CKS and HS, respectively. No significant associations were observed between the *NSMF* CNV genotype and withers height, body length, chest girth, and body weight in CKS (*p*-value > 0.05) (Table 2). However, the *NSMF* CNV significantly affected cannon circumference (*p*-value = 0.02) in HS, but showed no significant effects on withers height, pin bone width, chest girth, and body weight (*p*-value > 0.05). Notably, the association with body diagonal length was marginally significant (*p*-value = 0.05) (Table 3). HS individuals with the normal genotype had a significantly greater cannon circumference than those with the deletion genotype, but no significant difference was observed compared to individuals with the duplication genotype. Individuals with the duplication genotype had significantly smaller body diagonal length than HS individuals with both the deletion and normal genotypes; however, there was no significant difference between the deletion and normal genotypes, although the normal genotype tended to have a larger body diagonal length than the deletion genotype. Overall, HS individuals with the normal genotype appeared to have larger body size and weight than those with the deletion and duplication genotypes.

## 4. Discussion

This study investigated the association between an *NSMF* CNV and growth traits in three Chinese sheep breeds (CKS, HS, and STHS). We validated a previously identified deletion CNV within the *NSMF* gene using qPCR and analyzed its genotype frequencies and associations with growth traits.

A key methodological aspect of this study was the use of the 2^−ΔCt^ method for copy number determination, rather than the more commonly used 2^−ΔΔCt^ method. A crucial prerequisite for employing the 2^−ΔΔCt^ method in quantitative analysis is the assumption of equivalent amplification efficiencies between the target and reference genes [24]. Given this prerequisite, we assume the existence of an individual with the normal genotype to be used as a calibrator to calculate ΔΔCt. In this case, the ΔCt of this calibrator individual would be 0, meaning the Ct value of the *NSMF* CNV in this individual is equal to the Ct value of the *ANKRD1* gene, which is widely accepted to be devoid of CNVs and have two copies in all individuals [22,23]. Therefore, 2^−ΔΔCt^ could be simplified to 2^−ΔCt^. If the 2^−ΔΔCt^ method was used, it would require the selection of a calibrator individual. However, given the high prevalence of deletions in our studied breeds, selecting a representative “normal” calibrator becomes challenging. Furthermore, using the 2^−ΔΔCt^ method, the calculated *NSMF* copy numbers would be influenced by the chosen calibrator, potentially leading to significant variations in the results. The 2^−ΔCt^ method has been successfully applied in copy number calculation [22,23].

After calculating *NSMF* copy numbers using the 2^−ΔCt^ method, experimental animals were classified into three groups based on rounded results: deletion (copy number < 2), normal (copy number = 2), and duplication (copy number > 2). The genotype frequencies of deletion, normal, and duplication in CKS were 90.57%, 3.37%, and 6.06%, respectively; in HS, they were 73.44%, 12.50%, and 14.06%, respectively; and in STHS, they were 98.44%, 1.56%, and 0%, respectively. The observed differences in the *NSMF* CNV genotype frequencies among the three breeds highlight their diverse genetic backgrounds. Chaka sheep, native to the Qinghai Plateau and adapted to its hypersaline environment and plateau habitat, are also known as Qinghai Plateau wool–mutton-type semi-fine-wool sheep [25]. This breed, which has been designated a geographical indication of agricultural products by the Chinese Ministry of Agriculture, was developed in Qinghai Province, China, in 1987 and has ancestry from Xinjiang fine-wool sheep, Tibetan sheep, and Romney sheep [26]. Hu sheep, a unique Chinese breed recognized for its exceptional prolificacy, is an excellent maternal breed utilized in meat sheep crossbreeding programs for genetic improvement [27]. This distinctive white pelt sheep breed, with origins tracing back to Mongolian sheep, has a history of over 1000 years of adaptation, transitioning from extensive grazing to intensive indoor management and thriving in the hot and humid conditions characteristic of the regions south of the Yangtze River of China [26]. The Small-tailed Han sheep, a dual-purpose breed utilized for both meat and pelt production, is predominantly distributed in the plains of the Yellow River [26]. This breed is characterized by high fecundity, with reported litter sizes ranging from 2.61 to 2.65 lambs [28]. Their management typically involves intensive indoor housing as the primary method, supplemented by grazing.

A significant finding of this study was the association between the *NSMF* CNV and growth traits in HS, specifically cannon circumference (*p*-value = 0.021) and a marginally significant association with body diagonal length (*p*-value = 0.050). This suggests that the *NSMF* gene, or a linked gene within the CNVR, may play a role in skeletal development and limb morphology in HS. The identification of the *NSMF* CNV as a potential marker for cannon circumference and body diagonal length in Hu sheep has direct relevance for breeding programs. The two growth traits are often associated with overall body size and muscling in sheep. By incorporating this CNV information into selection strategies, breeders could potentially identify individuals with a genetic predisposition for larger body size, leading to faster genetic progress for important traits. This could result in improved meat production and carcass quality in Hu sheep.

It is indeed documented in the literature that *NSMF* gene CNVs are associated with synkinesia, scoliosis, and bone abnormalities in humans [29]. Nevertheless, this *NSMF* CNV does not appear to influence growth traits in CKS. This absence of phenotypic effect may be attributed to breed-specific genetic backgrounds. The mechanisms by which CNVs influence gene expression are diverse, including gene dosage effects (where changes in copy number directly alter the amount of gene product), gene interruption (where CNVs disrupt the gene sequence, leading to loss of function), and gene fusion (where CNVs fuse parts of different genes, creating novel chimeric genes) [30]. However, these established mechanisms appear insufficient to explain the observed lack of phenotypic effect of the *NSMF* CNV in CKS. It is important to consider that CNVs can also exert position effects. This refers to the phenomenon where a CNV alters a gene’s location on the chromosome, potentially translocating it in proximity to regulatory elements [31]. These regulatory elements often exhibit spatiotemporal specificity and result in CNVs having breed-specific phenotypic consequences [32]. Additionally, it is possible that breed-specific single-nucleotide polymorphisms (SNPs) exist within the *NSMF* CNV region. These SNPs could exhibit significant differential allelic effects on transcription factor-binding sites, influencing the binding affinity of transcription factors and consequently affecting gene expression [33]. This mechanism may also contribute to the different phenotypic effects of the *NSMF* CNV in CKS and HS.

## 5. Conclusions

In conclusion, our study identified a significant association of the *NSMF* CNV with body diagonal length and cannon circumference in HS. However, further studies with a larger HS cohort are crucial to validate these findings and definitively establish the *NSMF* CNV as a reliable molecular marker for HS breeding and selection programs.

## Figures and Tables

**Table 1:** Primer sequences used in this study.

Primer ID	Sequences (5′-3′)	Products (bp)
NSMF-F	GGCTACATCTGGGACCATCTT	137
NSMF-R	CGAGCCGCCCAATTTTATGGT	
ANKRD1-F	AAGACCCCCGAAATGCTACC	128
ANKRD1-R	GCCGACCTCAACGTCAAGAA	

**Table 2:** Association between *NSMF* CNV genotypes with growth traits of CKS.

Growth Traits	CNV Types (LSM ± S.E.)			*F*-Value	*p*-Value
	Deletion (n = 269)	Normal (n = 10)	Duplication (n = 18)		
Withers height (cm)	66.11 ± 0.26	66.04 ± 1.34	66.16 ± 1.00	0.00	1.00
Body length (cm)	72.00 ± 0.42	71.20 ± 2.16	72.28 ± 1.61	0.08	0.92
Chest girth (cm)	89.31 ± 0.49	91.08 ± 2.54	91.74 ± 1.90	0.97	0.38
Body weight (kg)	53.99 ± 0.77	56.00 ± 3.97	57.00 ± 2.97	0.59	0.56

CNV, copy number variation; CKS, Cha Ka sheep; LSE, least square means; S.E., standard error.

**Table 3:** Association between NSMF CNV genotypes with growth traits of HS.

Growth Traits	CNV Types (LSM ± S.E.)			*F*-Value	*p*-Value
	Deletion (n = 47)	Normal (n = 8)	Duplication (n = 9)		
Withers height (cm)	64.98 ± 0.42	66.00 ± 0.96	65.56 ± 1.09	0.51	0.60
Pin bone width (cm)	17.14 ± 0.23	16.94 ± 0.63	16.44 ± 0.47	0.74	0.48
Body diagonal length (cm)	70.66 ± 0.48 a	71.75 ± 1.21 a	67.56 ± 2.04 b	3.16	0.05
Chest girth (cm)	76.60 ± 0.50	78.00 ± 2.04	79.56 ± 1.64	2.29	0.11
Cannon circumference (cm)	7.20 ± 0.06 b	7.63 ± 0.18 a	7.50 ± 0.22 ab	4.14	0.02
Body weight (kg)	34.02 ± 0.61	34.63 ± 0.72	32.92 ± 1.48	0.42	0.66

Different letters in the same row mean significant difference (a, b: *p* < 0.05). CNV, copy number variation; HS, Hu sheep; LSE, least square means; S.E., standard error.

## Data Availability

The original contributions presented in the study are included in the article, further inquiries can be directed to the corresponding author.

## Abbreviations

CNV	copy number variation
qPCR	quantitative PCR
CKS	Chaka sheep
HS	Hu sheep
STHS	Small-tailed Han sheep
